# STAT4 gene polymorphism in two major autoimmune diseases (multiple sclerosis and juvenile onset systemic lupus erythematosus) and its relation to disease severity

**DOI:** 10.1186/s41983-018-0011-5

**Published:** 2018-05-25

**Authors:** Rania S. Nageeb, Alaa A. Omran, Ghada S. Nageeb, Manal A. Yousef, Yassir A. A. Mohammad, Amal Fawzy

**Affiliations:** 1 Department of Neurology, Faculty of Medicine, Zagazig University, Sharkia, Egypt; 2 Department of Clinical Pathology, Faculty of Medicine, Zagazig University, Sharkia, Egypt; 3 Department of Rheumatology and Rehabilitation, Faculty of Medicine, Zagazig University, Sharkia, Egypt; 4 Department of Chemistry, Faculty of Medicine, Zagazig University, Sharkia, Egypt

**Keywords:** STAT4, Multiple sclerosis (MS) and juvenile onset systemic lupus erythematosus (JO-SLE)

## Abstract

**Background:**

Multiple sclerosis (MS) and systemic lupus erythematosus (SLE) are chronic autoimmune mediated diseases with strong genetic and environmental components. The aim of this study is to evaluate the association of STAT4 gene polymorphism with multiple sclerosis (MS) and juvenile onset systemic lupus erythematosus (JO-SLE) and its relation to disease severity.

**Methods:**

Group 1 consisted of 40 MS patients while group 2 included 40 JO-SLE patients. Forty healthy volunteers (controls) were included in this study. STAT4 genotyping was performed by polymerase chain reaction-restriction fragment length polymorphism (PCR-RFLP).

**Results:**

The STAT4 CC genotype and GC genotype frequencies were significantly more detected in MS and JO-SLE patients than in controls. The frequency of the STAT4 C allele was significantly higher in patients with MS and those with JSLE compared to controls. Malar rash, photosensitivity, and hair falling were significantly more detected in CC subtype. Malar rash, photosensitivity, and hair falling were significantly more detected in CC subtype. Increased 24-h protein in urine (mg/24 h) and ANA positivity, anti-ds-DNA, anti Sm antibodies’ detection and decreased C3 and C4 levels showed a significantly difference in CC patients. Meanwhile, only increased 24-h protein in urine (mg/24 h) and ANA positivity were significantly more detected in GC patients. STAT4 CC genotype showed a significant increase in the SLE activity index (SLEAI) score and damage index as compared to the STAT4 GG genotype patients. No significant difference was detected in MS Kurtzke’s Expanded Disability Status Scale (EDSS) comparing different STATE 4 genotypes.

**Conclusions:**

STAT4 polymorphism was significantly associated with MS and JO-SLE. Though homozygous JO-SLE patients are more risky for severe disease manifestations, homozygous MS patients are not risky for severe disease disability.

## Background

Multiple sclerosis (MS) and systemic lupus erythematosus (SLE) are chronic autoimmune diseases with strong genetic and environmental components. They can be triggered by various environmental components, such as exposure to ultraviolet light, drugs, chemicals, and viral infections. SLE disease is characterized by the presence of pathogenic auto-antibodies against a number of nuclear antigens, which results in immunologic abnormalities and multiple organ damage (Piotrowski et al. 2012; Prahalad et al. 2009).

The course of MS disease is categorized based on how clinical manifestations develop over time and on the severity of the disease (Anlar 2009). Systemic lupus erythematosus disease severity is wide ranging, from mild forms to fatal depending on organ involvement (Kawasaki et al. 2008; Ben-Menachem 2010).

The human STAT (signal transducer and activator of transcription) genes have been identified in three chromosomal clusters: STAT1 and STAT4 on human chromosome 2 (q12-33); STAT2 and STAT6 on chromosome 12 (q13-14); and STAT3, STAT5a, and 5b on chromosome 17 (q11.2-22). This gene encodes a transcription factor that can be activated by interleukin (IL)-12 and IL-23 and plays a role in the signaling via type-1 interferon (IFN I) receptor (Ceccarelli et al. 2015). Genome studies had identified STAT4 as a susceptible SLE gene in Caucasian and Asian populations (Taylor et al. 2008). STAT4 is essential for IL-12 signaling and induces interferon-gamma (IFNγ) production (Gestermann et al. 2010).

Signal transmission from the interferons involves STAT1 and STAT4 (Raafat et al. 2015). The extensive involvement of type I and type II interferons in the pathogenesis of MS and SLE made STAT4 an obvious candidate region for genetic predisposition to these autoimmune diseases (Bolin et al. 2013). Moreover, the requirement of STAT4 in IL-23-induced IL-17 production has been suggested (Tanasescu et al. 2010).

We hypothesized that STAT4 (rs7582694) gene polymorphism contribute to autoimmune diseases. We therefore studied its polymorphism in multiple sclerosis (MS) and juvenile onset systemic lupus erythematosus (JO-SLE) and its relation to disease severity.

## Methods

All MS and JO-SLE patients attending Zagazig University Hospitals as well as insurance hospitals in Sharkia, governorates, Egypt, in the period from January 2015 to July 2017 were included in the current study. The study included two groups of patients: group 1 (included 40 MS patients) and group 2 (included 40 JO-JSLE patients) with an average age (mean ± SD)/years 29.29 ± 8.47and 19.09 ± 3.67, respectively.

### Ethical consideration

Informed consent was taken from all of the subjects or their relatives before enrollment. The study was approved by the Institutional Ethical Committee of Faculty of Medicine, Zagazig University, Egypt.

#### Inclusion criteria:

Group 1 was diagnosed to have definite MS according to the McDonald criteria (Polman et al. 2011). Group 2 was diagnosed according to the Systemic Lupus International Collaborating Clinics classification criteria for systemic lupus erythematosus (SLE) (Petri et al. 2012). Adults with an age at disease onset starting before 16 years were grouped as JO-SLE. Control group included 40 apparently healthy volunteers; ages and sex matched with patient groups. Their age (mean ± SD)/years were 25.19 ± 12.57 and 14.45 ± 3.33, respectively. They were recruited from relatives of the patients and individuals attending for blood donation.

#### Exclusion criteria: other autoimmune diseases.

All patients were subjected to the following: complete history taking (with special stress on age, gender, duration of disease, and family history) and clinical examination. Multiple sclerosis disability was quantified using Kurtzke’s Expanded Disability Status Scale (EDSS). The EDSS provides a total score on a scale that ranges from 0 to 10. The first levels 1.0 to 4.5 refer to people with a high degree of ambulatory ability, and the subsequent levels 5.0 to 9.5 refer to the loss of ambulatory ability (Kurtzke 1983). All MS cases underwent brain MRI after triple dose gadolinium diethylene-triaminepenta-acetic acid. For JO-SLE patients, the SLE activity index (SLEDAI) was used to assess disease activity (Galdman et al. 2000) and Systemic Lupus International Collaborating Clinics/American College of Rheumatology damage index (SLICC/ACR) was used to assess accumulated damage (Gladman et al. 1997). All participants underwent laboratory investigations such as complete blood count, ESR, liver and kidney functions, routine urine examination, and 24-h urinary protein. C3 and C4 assessment, antinuclear antibodies, and double-stranded DNA (ds-DNA) antibodies by immunofluorescence method (Diasorin, USA) and anti-sm, anti-SSA (Ro), anti-SSB (La), anti-RNP, anti-SCL 70, and anti Jo1 were detected by enzyme-linked immunosorbent assay (ELISA) in JO-SLE patients.

#### Genotyping of MS and JO-SLE patients and controls:

**Fig. 1:**
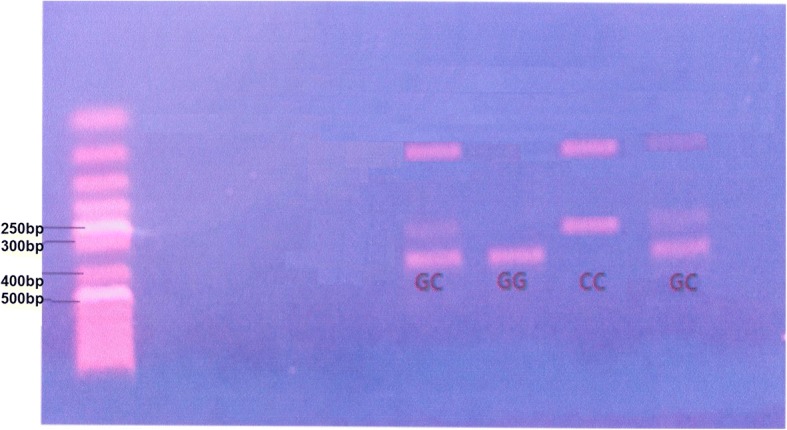
Restriction fragment length polymorphism-polymerase chain reaction (RFLP-PCR) analysis of STAT4 gene polymorphism in multiple sclerosis patients

**Fig. 2:**
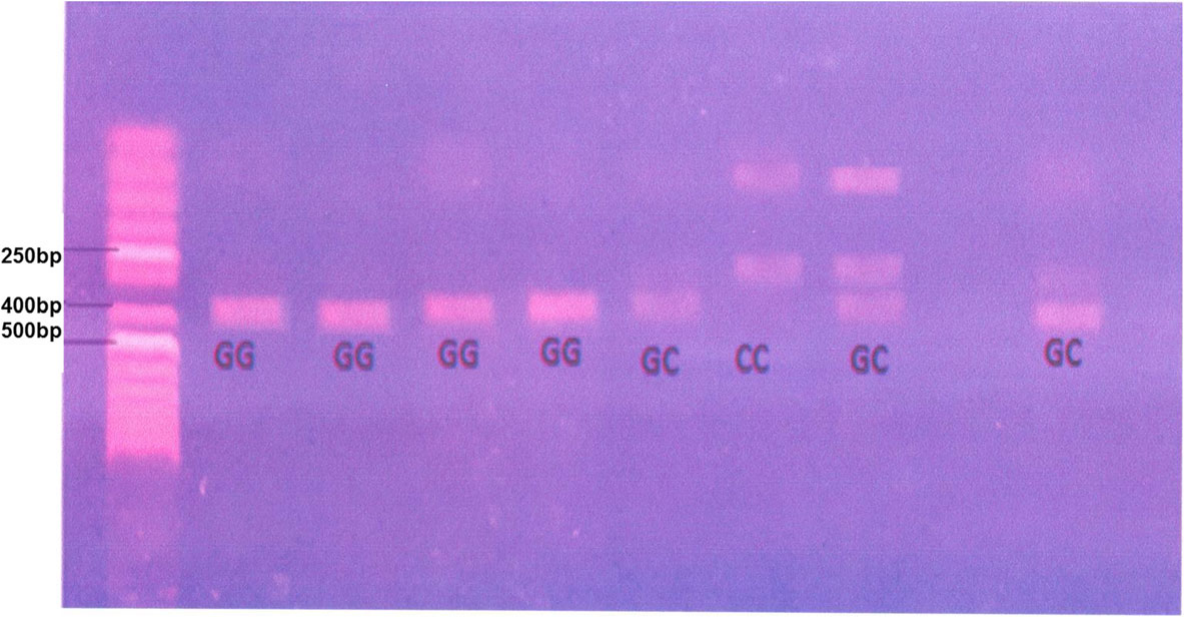
Restriction fragment length polymorphism-polymerase chain reaction (RFLP-PCR) analysis of STAT4 gene polymorphism in juvenile onset systemic lupus erythematosus patients (JO-SLE)

Genomic DNA was extracted from peripheral blood leukocytes of all participants using a QIA Amp DNA Minikit (Qiagen) according to manufacturer’s instructions. Identification of the STAT4 (rs7582694) polymorphic was performed by using polymerase chain reaction-restriction fragment length polymorphism (PCR-RFLP). PCR was conducted employing primer pair. Forward primer was 5′ ATCCAACTCTTCTCAGCCCTT 3′, and reverse primer was 5′ TCATAATCAGGAGAGAGGAGT 3′. The PCR-amplified fragments of STAT4 (338 bp in length) were isolated and digested with endonuclease TAAl (Sigma). DNA fragments were separated by agarose gel electrophoresis then visualized by ethidium bromide staining (Figs. 1 and 2).

### Statistical analysis

Parametric data were expressed as mean, standard deviation, while non-parametric data were expressed as median. Chi-square test or Fisher’s exact test was used to detect the relation between two qualitative variables (post hoc chi-square was used comparing three). *T* test and analysis of variance (ANOVA) were used to detect the relation between quantitative variables (comparing two groups and three groups respectively). Logistic regression analysis was used to calculate odds ratios (OR) and 95% confidence intervals (CI) for risk estimation. *P* values less than 0.05 were considered significant (Levesque 2007).

## Results

### Characteristic of the patients

This study included 40 MS patients (group 1) and 40 JO- SLE patients (group 2). A control group composed of 40 apparently healthy volunteers that were age and sex matched with the patients were included in the study. The disease duration was 6.1 ± 4.54 years and 8.1 ± 1.54 years in group 1 and group 2, respectively.

In the current study. 11 (27.5%) MS patients presented with progressive course and 29 (72.5%) patients with relapsing remittent (RR) MS with mean Expanded Disability Status Scale (EDSS) score of patients was 4.76 ± 2.65. Regarding the number of attacks, 52.5% of patients had ≤ 3 attacks and 47.5% of patients had > 3 attacks.

Regarding the age and sex, there were no significant difference between patients and their controls (Table 1).

**Table 1:** Demographic data of all of the studied groups

Variable		Patients (group 1)	Patients (group 2)	Controls
		MS patients No = 40	JO-JSLE patients No = 40	No = 40
Age/years		29.29 ± 8.47	21.09 ± 3.67	25.19 ± 12.57
Gender	Females	34(85%)	37(91.5%)	33(82.5%)
	Males	6(15%)	3(8.5%)	7(17.5%)
Disease duration/years		6.1 ± 4.54	8.1 ± 1.54	–

Quantitative parametric data are represented in mean (±SD), and numerical data are represented in numbers and percentages

*MS* multiple sclerosis, *JO-JSLE* juvenile onset systemic lupus erythematosus

The STAT 4 CC genotype and GC genotype frequencies were significantly more detected in MS and JO-SLE patients than in controls (*p* = 0.01 and 0.05 and *p* = 0.001 and 0.01 for both respectively). The frequency of the STAT4 C allele was significantly higher in patients with MS and those with JO-SLE than in controls (*p* = 0.01 and 0.001 respectively), (Table 2).

**Table 2:** Genotypes of all of the studied groups

	Frequency		Patients	Controls	CI	OR	*P* value
Group 1	Genotype	CC	5(15%)	3(7.5%)	5.15 (1.13–2.3)	13.19	0.01*
		GC	15(37.5%)	12(30%)	1.4 (0.52–0.92)	9.74	0.05*
		GG	20(50%)	25(62.5%)	0.9 (0.1–1.2)	7.2	0.06
	Allele	G	17(42.5%)	30(75%)	1.9 (0.3–1.5)	6.5	0.20
		C	23(57.5%)	10(25%)	8.9 (8.71–14.89)	1. 72	0.01*
Group 2	Genotype	CC	7(17%)	2(4%)	6.95 (12.17–22.3)	12.89	0.001*
		GC	17(42%)	10(25%)	2.9 (1.52–5.52)	10.74	0.01*
		GG	16(41%)	28(71%)	1.3 (0.3–2.5)	6.8	0.18
	Allele	G	15(38.3%)	33(82.97%)	1.6 (0.1–1.4)	7.7	0.42
		C	25(61.7%)	7(17.2%)	3.1 (1.87–4.89)	21.27	0.001*

Multiple logistic regression analysis was used. Numerical data are represented in numbers and percentages

*Significant

Comparing both CC and GC patients versus GG and comparing both CC versus GC showed that there were no significant difference between clinical parameters of MS patients and genotypic pattern (Table 3).

**Table 3:** Relation between clinical parameters of MS patients and genotypic pattern

Variable		CC (*n* = 7)	GC (*n* = 17)	GG (*n* = 16)	P value
Age/years		19.44 ± 7.25	20.13 ± 7.08	19.5 ± 4.4	0.4
Gender	Female	6(86%)	14(82%)	14(88%)	0.22
	Male	1(14%)	3(18%)	2(0.18)	0.31
Disease duration/years		5.41 ± 2.32	5.47 ± 3.25	5.11 ± 2.32	0.12
Disease course	Progressive	4(57%)	4(24%)	3(19%)	0.4
	RR	3(43%)	13(76%)	13(81%)	0.22
EDSS	< 6	3(43%)	5(29%)	6(37%)	0.61
	≥ 6	4(57%)	12(71%)	10(63%)	0.12
Number of attacks	< 3	4(57%)	9(53%)	8(50%)	0.5
	≥ 3	3(43%)	8(47%)	8(50%)	0.2

Chi-square and post hoc chi-square for qualitative data and ANOVA for quantitative data. Numerical data are represented in numbers and percentages

*RR* relapsing-remitting, *EDSS* Expanded Disability Status Scale

Comparing both CC and GC patients versus GG in JO-SLE patients showed that patient’s malar rash, photosensitivity, and hair falling were significantly more detected in the CC subtype. Comparing both CC versus GC showed that patient’s malar rash, photosensitivity, and hair falling were significantly more detected in the CC subtype (Table 4).

**Table 4:** Relation between clinical parameters of JO-SLE patients and genotypic pattern

Variable	CC (*n* = 7)	GC (*n* = 17)	GG (*n* = 16)	*P* value
Age/years	20.44 ± 7.25	19.13 ± 7.08	19.6 ± 4.8	0.9
Disease duration/years	5.41 ± 2.23	6.74 ± 3.25	5.12 ± 2.25	0.4
Fever	2(29%)	4(24%)	3(19%)	0.11
Malar rash	3(43%)*^+^	5(29%)	6(38%)	0.01
Photosensitivity	4(57%)*^+^	3(18%)	3(19%)	0.02
Arthritis	2(29%)	4(24%)	4(25%)	0.2
Serositis	2(29%)	4(24%)	4(25%)	0.3
Hematological	4(57%)	10(59%)	10(63%)	0.3
Hair falling	3(43%)*^+^	6(35%)	6(38%)	0.04
Neurologic affection	2(29%)	4(24%)	3(19%)	0.11
Vacuities	2(29%)	4(24%)	3(19%)	0.2

Chi-square and post hoc chi-square for qualitative and ANOVA for quantitative data. Quantitative parametric data are represented in mean (±SD) and numerical data are represented in numbers and percentage

*Significant comparing either the first or second column versus third column

^+^Significant comparing the first column versus second column

Increased 24-h protein in urine (mg/24 h) and ANA positivity, anti-ds-DNA, anti Sm antibodies’ detection and decreased C3 and C4 levels showed a significantly difference in CC patients. Meanwhile, only increased 24-h protein in urine (mg/24 h) and ANA positivity were significantly more detected in GC patients (Table 5).

**Table 5:** Comparing laboratory findings in patients with JO-SLE according to STAT4 polymorphic variants (CC, GC, and GG)

Variable	CC (*n* = 7)	GC (*n* = 17)	GG (*n* = 16)	*P* value
24-h protein in urine(mg/24 h)	1346 ± 616*	1189 ± 215*	698 ± 807	0.02
C3 level (mg/dl)	31.34 ± 11.8*^+^	51.32 ± 17.55	58.1 ± 14.5	0.01
C4 level (mg/dl)	6.61 ± 2.08*^+^	13.3 ± 2.88	16.9 ± 3.95	0.03
Anti-ds-DNA	6(86%)*	9(53%)	8(50%)	0.01
ANA	7(100%)*	17(100%)*	14(88%)	0.02
Ant SS-A	2(29%)	5(29%)	7(44%)	0.3
Anti SS-B	0	0	3(19%)	0.5
Anti Sm	3(43%)*^+^	2(12%)	3(19%)	0.04
Anti RNP	1(14%)	3(18%)	3(19%)	0.11
Anti Scl 70	1(14%)	2(12%)	2(13%)	0.8
Anti Jo-1	1(14%)	0	0	0.9

Chi square and post hoc chi-square for qualitative and ANOVA for quantitative data. Quantitative parametric data are represented in mean (±SD) and numerical data are represented in numbers and percentage

*Significant comparing either the first or second column versus third column

^+^Significant comparing the first column versus second column

**Fig. 3:**
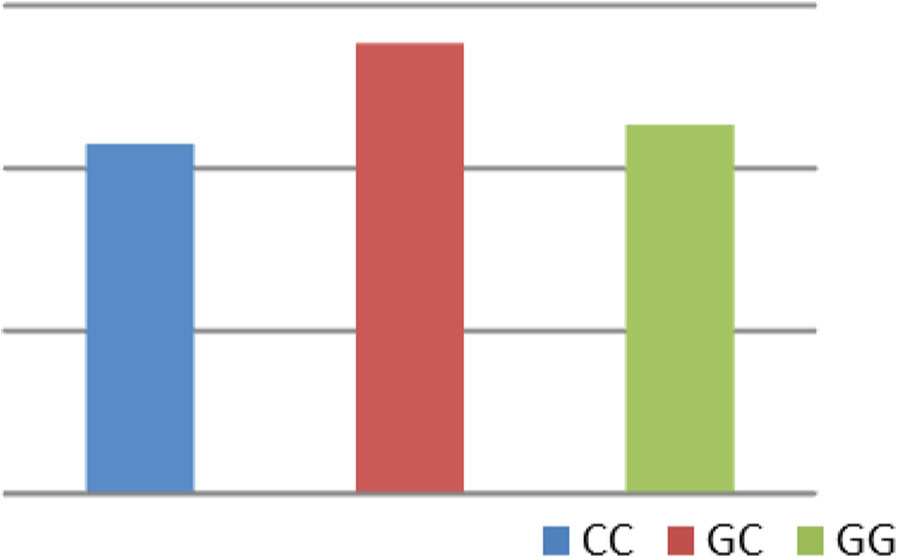
Association of STAT4 polymorphism and Kurtzke’s Expanded Disability Status Scale (EDSS) of group 1 (multiple sclerosis)

No significant difference was detected in Kurtzke’s Expanded Disability Status Scale (EDSS) comparing different STATE 4 genotypes (Fig. 3).

**Fig. 4:**
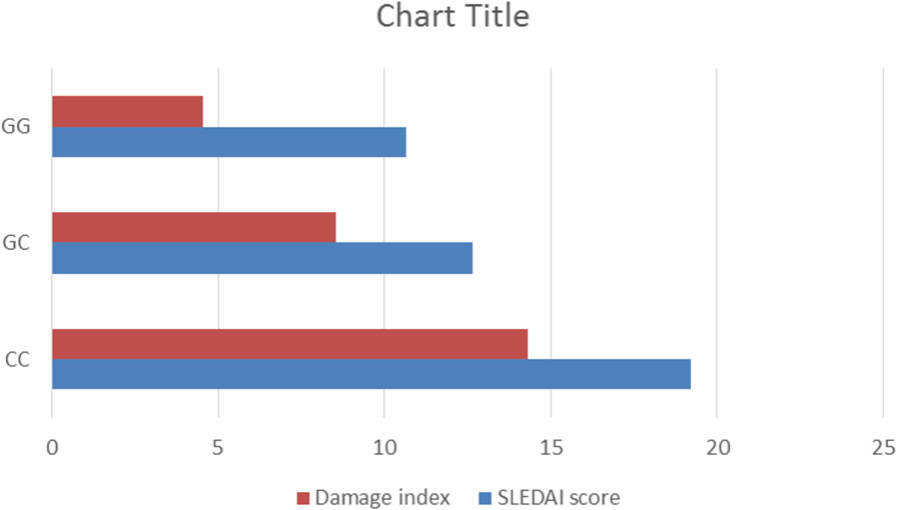
Association of STAT4 polymorphism with SLE activity index (SLEDAI score) and Systemic Lupus International Collaborating Clinics/American College of Rheumatology damage index (SLICC/ACR) in group 1 (SLE patients)

STAT4 CC genotypes showed a significant increase in SLEAI and damage index as compared to other STAT4 genotype patients (Fig. 4).

## Discussion

The scope of this study was to study the association of STAT4 (rs7582694) gene polymorphism with two autoimmune diseases, e.g., multiple sclerosis (MS) and juvenile onset systemic lupus erythematosus patients (JO-SLE) and its relation to disease severity.

The STAT 4 CC genotype and GC genotype frequencies were significantly more detected in MS patients than in controls (*p* = 0.01 and 0.05 respectively). The frequency of the STAT4 C allele was significantly higher in patients with MS than in controls (*p* = 0.01). There were no significant difference between clinical parameters of MS patients and genotypic pattern. Meanwhile, no significant difference was detected in Kurtzke’s Expanded Disability Status Scale (EDSS) comparing different STATE 4 genotypes (*p* = 0.07).

The present findings met with the findings of another study conducted on MS patients that demonstrated that IL-17 F CT genotype and C allele may be associated with a susceptibility to MS in Egyptian population by a gender-dependent mechanism that contributes to unique predisposition in females. So, this rs763780 can be considered a risk factor for the development of MS in the Egyptian population (Atya et al. 2017).

In contrary, Liang et al. (2012) demonstrated no association between the STAT4 rs7574 865T allele and MS. Since the subgroup of analysis for multiple sclerosis in their study included no more than five studies for the meta-analysis, they could not draw funnel plots for each of them. This may not have enough power to explore the association between the STAT4 rs7574865 single-nucleotide polymorphism (SNP) and MS.

The STAT 4 CC genotype and GC genotype frequencies were significantly more detected in JO-SLE patients than in controls (*p* = 0.001 and 0.01 respectively). The frequency of the STAT4 C allele was significantly higher in patients with JO-JSLE than in controls (*p* = 0.001). That met with findings of another study (Taylor et al. 2011); they found that the SNP rs7574865 of STAT4 resulted in being associated with younger age at diagnosis (OR = 1.22). Also, Liang et al. (2012) demonstrated a statistically significant contribution of STAT4 to juvenile SLE incidence in the mainland Chinese female population. The frequency of the same genetic variant resulted in being slightly higher in SLE Japanese patients with an age of onset lower than 20 years as compared with patients with age ≥ 20 years (El-Saadany et al. 2016). Another study (Zhou et al. 2014) demonstrated a statistically significant contribution of STAT4 to juvenile idiopathic arthritis.

In addition, Hammad et al. (2017) found that rs2004640 T allele and TT genotype and GTA haplotype of rs 10954213, rs2004640, and rs2280714, respectively, can be considered as risk factors for the development of SLE in Egyptian children. The presence of the rs2004640 T allele increases the risk of development of nephritis in Egyptian children with SLE.

On the other hand, Raafat et al. (2015) found that STAT4 polymorphism was not associated with an increased risk of SLE in Egyptian females; this may be attributed to the difference in the method of genotyping that was performed by the real-time PCR allelic discrimination technique in the previous study.

In the present study, comparing both CC and GC patients versus GG in JO-SLE patients showed that patient’s malar rash, photosensitivity, and hair falling were significantly more detected in the CC subtype. Also, comparing both CC versus GC showed that patient’s malar rash, photosensitivity, and hair falling were significantly more detected in the CC subtype. Other studies reported the significant association between the SNP rs7574865 of STAT4 genotypes and the presence of photosensitivity (Taylor et al. 2008).

Increased 24-h protein in urine (mg/24 h) and ANA positivity, Anti ds-DNA, anti Sm antibodies’ detection and decreased C3 and C4 levels showed a significantly difference in CC patients of the present study. Meanwhile, only increased 24-h protein in urine (mg/24 h) and ANA positivity were significantly more detected in GC patients.

Other studies determined the strong association between STAT4 rs7574865 polymorphism and with severity of SLE. Piotrowski et al. (2012) found that SNP was significantly associated with more badly renal symptoms in SLE. Also, Taylor et al. (2011) agreed that for SLE patients, C allele in rs7574865 correlated with proteinuria, C3 and C4 levels, and anti-dsDNA positivity. On the other hand, El-Saadany et al. (2016) found that the C allele or CC homozygous is a significant risk genetic molecular marker to predict SLE susceptibility but they found no association for lupus nephritis. Possible explanations for these findings might be the difference in ethnic groups.

The present study showed that JO-SLE patients with a CC homozygous had higher SLEAI and damage index than with other genotypes. This is in accordance with Raafat et al. (2015) and Taylor et al. (2011)) Interestingly, Bolin et al. (2013) reported the significant association of STAT4 CC polymorphism with severe renal insufficiency in lupus nephritis.

## Conclusions

STAT4 polymorphism was significantly associated with multiple sclerosis (MS) and juvenile onset systemic lupus erythematosus patients (JO-SLE). Though homozygous JO-SLE patients are more risky for severe disease manifestations, homozygous MS patients are not risky for severe disease disability.

## Abbreviations

ds-DNA: Double-stranded DNA
EDSS: Expanded Disability Status Scale
ELISA: Enzyme-linked immunosorbent assay
IFN I: Type-1 interferon
IFNγ: Interferon-gamma
JO-SLE: Juvenile onset systemic lupus erythematosus
MS: Multiple sclerosis
PCR-RFLP: Polymerase chain reaction-restriction fragment length polymorphism
SLE: Systemic lupus erythematosus
SNP: Single-nucleotide polymorphism
STAT: Signal transducer and activator of transcription
